# Daphnane diterpene hirsein B downregulates melanogenesis in B16 murine melanoma cells by cAMP pathway inhibition

**DOI:** 10.1186/1753-6561-5-S8-P68

**Published:** 2011-11-22

**Authors:** Myra O Villareal, Junkyu Han, Kenjiro Ikuta, Hiroko Isoda

**Affiliations:** 1 Graduate School of Life and Environmental Sciences, University of Tsukuba, Tennodai 1-1-1, Tsukuba, Ibaraki, 305-8572, Japan; 2 Alliance for Research on North Africa (ARENA), University of Tsukuba, Tennodai 1-1-1, Tsukuba, Ibaraki, 305-8572, Japan; 3 Yokohama Corporate Research Laboratories, Mitsubishi Rayon Co., Ltd, Tsurumi-ku, Yokohama, Kanagawa, 230-0053, Japan

## Background

Skin pigmentation serves as protection against ultraviolet-induced skin damage through melanin’s optical and chemical filtering properties [1]. Although melanin plays and important role in skin protection, excessive melanin production or hyperpigmentation may lead to skin cancer. Recently, the inhibition of melanogenesis has been considered as a valid therapeutic target for the management of advanced melanotic melanomas [2] which increases the need for melanogenesis inhibitors that are of plant origin and are not cytotoxic to mammalian cells. The biosynthesis of the pigment melanin is catalyzed by the melanogenic enzymes tyrosinase, tyrosinase related protein 1 and the dopachrome tautomerase, the transcriptional regulation of which is being regulated by the microphthalmia associated transcription factor (*Mitf*) [3]. Previously, we have reported that hirsein B (HB) or 5β-hydroxyresiniferonol-6α,7α-epoxy-12β-coumaroyloxy-9,13,14-*ortho*-decanoate from *Thymelaea hirsuta *[4] has antimelanogenesis effect (without cytotoxicity) on B16 murine melanoma cells by downregulating the expressions of the *Mitf* gene and the melanogenic enzymes’ genes [5]. The exact mechanism by which hirsein B inhibited the *Mitf* gene expression, however, has not yet been determined. In melanogenesis, the *Mitf* gene expression can be regulated through the cAMP pathway or the Wnt signaling pathway. This study aimed to determine the mechanism underlying the inhibitory effect of HB on *Mitf* gene in B16 murine melanoma cells.

## Materials and methods

Total RNA was isolated from B16 murine melanoma cells (Riken Cell Bank, Tsukuba, Japan) and used for DNA microarray analysis, using chips of 528 spots loaded with 265 genes prepared by Genopal™ (Mitsubishi Rayon Co., Ltd, Tokyo, Japan), to determine the expressions of genes for melanogenesis, membrane-bound receptors, tyrosine kinase regulation, melanosome transport, and other cell signal regulation-related genes (including the housekeeping and negative control genes). To validate the results, real-time PCR, using TaqMan FAST 7500 (Applied Biosystems, Foster City, CA, USA) and specific TaqMan primers (Applied Biosystems, Foster City, CA, USA) for the differentially-expressed genes, was performed.

## Results

Results showed that the expressions of the *Mitf* gene and the melanogenic enzymes’ genes were downregulated, verifying our previous report [5]. In addition, the expression of the gene for melanocortin 1 receptor (*Mc1r*) of the cAMP pathway was downregulated while most of the genes that were upregulated are those involved in the Wnt signaling pathway (Table 1).

**Table 1:** Differentially-expressed genes in hirsein B-treated B16 murine melanoma cells as determined by DNA microarray.

Biological Process	Differentially expressed genes	Up- or Down- regulated
Melanin biosynthesis	*Mitf*, *Mc1r*	Downregulated
Melanosome transport	*Rab27a*, *Mlph*, *Myo5A*, *Myo7A*	Downregulated
Negative regulation of transcription from RNA polymerase II promoter	*Sorbs3*	Downregulated
Wnt signaling pathway	*Ppap2b*, *Wisp*, *Kremen1*	Upregulated
Cell cycle regulation	*Gadd45b*, *Csnkl*	Upregulated
Activation of MAPK signaling pathway	*Gadd45b* , *Pxn*, *Map2k3*, *Met*, *Avpi1*, *Spag9*	Upregulated
Cytoskeleton organization	*Pxn*	Upregulated
Protein phosphorylation	*Mapkapk3*	Upregulated

In mouse, peptide hormones from the pituitary gland bind to the *MC1R* and stimulate melanin production through the cAMP/PKA signalling pathway [6], by inducing changes in the protein phosphorylation and gene expression, through the MITF gene product.

## Conclusions

The results obtained suggest that the significant antimelanogenesis effect of hirsein B is through the inhibition of the expression of the *Mc1r* gene of the cAMP pathway. HB may therefore be used as a treatment for hyperpigmentation due to its significant melanogenesis downregulation effects in B16 cells or as a pretreatment for melanotic melanomas.
